# Diabetes and all-cause mortality, a 18-year follow-up study

**DOI:** 10.1038/s41598-020-60142-y

**Published:** 2020-02-21

**Authors:** Rezvan Salehidoost, Asieh Mansouri, Massoud Amini, Sima Aminorroaya Yamini, Ashraf Aminorroaya

**Affiliations:** 1 Isfahan Endocrine and Metabolism Research Center, Isfahan University of Medical sciences, Isfahan, Iran; 2 Hypertension Research Center, Cardiovascular Research Institute, Isfahan University of Medical Sciences, Isfahan, Iran; 3 Department of Engineering and Mathematics, Sheffield Hallam University, Sheffield, S1 1WB UK

**Keywords:** Diabetes complications, Type 2 diabetes

## Abstract

This study compared mortality rates and decline in life expectancy of Iranian patients with type 2 diabetes (T2DM) with the general population. A retrospective study of 2451 patients with T2DM was conducted in the Isfahan Endocrine and Metabolism Research Center, Iran, between 1992 and 2010. The mean (SD) of diabetes duration and median (Q1,Q3) of follow-up period were 15.5(8.0) and 8(5, 10) years. The main outcome was all-cause mortality. 732(29.87%) of patients died during the follow-up. Overall mortality rates (95%CI) per 1000 person-years in men and women were 56.3(52.0–62.1) and 27.3(24.5–30.4), respectively. The relative risks (95%CI) of all-cause mortality in males vs. females with T2DM aged 45–49, 50–54, 55–59, 60–64, 65–69, 70–74 were [3.02(1.49–6.11) vs. 2.09(0.96–4.57)], [4.05(2.73–6.01) vs. 2.29(1.52–3.45)], [4.13(3.26–5.24) vs. 1.70(1.23–2.35)], [2.42(1.90–3.07) vs. 1.82(1.46–2.27)], [2.36(2.02–2.76) vs. 1.49(1.25–1.78)] and [1.71(1.50–1.95) vs. 1.04(0.88–1.23)] times more than the general population, respectively. Men and women living with diabetes lost an average of 13.2(6.3) and 13.9(6.0) life-years from the year of diagnosis, respectively (p = 0.101). The estimated life-years lost were greater in younger patients and a gradual decline was observed with increasing the age at diagnosis. In conclusion, Iranians with diabetes had higher risk of death and lower life expectancy compared to the general population.

## Introduction

Type 2 diabetes mellitus (T2DM) is one of the most common health issues. There is a growing prevalence of T2DM, worldwide. In general, diabetes and its complications are leading causes of mortality in most countries^1–4^. Approximately 5.0 million deaths in 2015 was attributed to people, 20–79 years of age with diabetes, equivalent to one death in every six seconds^1^. The global all-cause mortality rate attributed to diabetes is 14.5% among people within this range of ages. This is higher than the combined number of deaths occurred by the infectious diseases^1^.

Studies have shown that the age-adjusted all-cause mortality in patients with diabetes is higher than the general population or people without diabetes^2,5–10^. Therisk of mortality tends to increase with diabetes duration and decreases with increasing age at which diabetes is diagnosed^2,6,7,9,10^. Lower life expectancy is reported for people with diabetes compared to people without diabetes^2,7,8^. However, majority of these studies were performed in Western populations^2,5–8,10^ and to our best of knowledge, there is no published data available for the excess risk of death and potential years of life lost in Iranian patients with T2DM.

The aim of the present study was to evaluate the mortality rates among people with diabetes compared to the general population of Iran according to age and sex. We also estimated the effect of diabetes on the expected life years by using clinical data registry records for patients with diabetes at the Isfahan Endocrine and Metabolism Research Center, Iran.

## Material and Methods

This retrospective database study was conducted in Isfahan, a large urban area located in the centre of Iran, with a population more than four and a half million (4,879,312 in 2011 (2,476,021 men and 2,403,291 women))^11^. The database of the Isfahan Endocrine and Metabolism Research Center was used to extract characteristic information of people with T2DM, registered in this center. Clinical data are collected for all patients by continuous enrollment at the first attendance and at follow up visits, which were performed with interval of 2 to 12 months. The demographic characteristics, medications and family medical history were recorded. The height, weight and blood pressure were measured and a general examination was performed at every visit. Fasting plasma glucose (FPG), HbA1c, triglyceride, cholesterol and other routine laboratory test were conducted for patients with diabetes. Patients’ data recording were continued from the baseline examination until they died or stopped the follow-ups. All patients were given informed consent for participation in the examination and registration.

**Figure 1:**
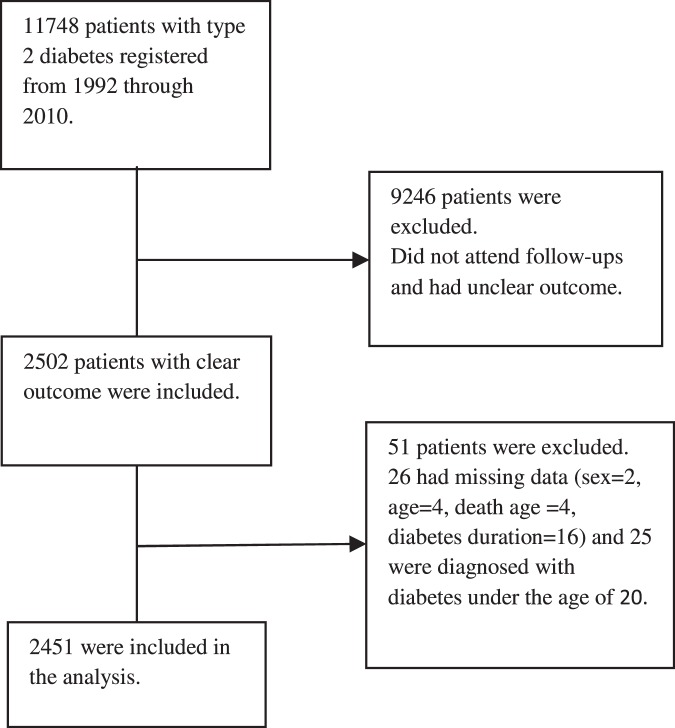
Selection process of participants.

Between year 1992 and 2010, a total number of 11748 patients with T2DM were registered in the system. However, this study only included 2502 of patients with T2DM who had follow-up visits and their survival status was known in 2010. Patients with missing data for sex (n = 2), age (n = 4), the age of death (n = 4) and the duration of diabetes (n = 16) or those patients who were diagnosed with diabetes at ages younger than 20 (most probably type 1 diabetes) (n = 25) were excluded from the analyses. Finally, a total number of 2451 (993 (40.51%) men and 1458 (59.49%) women) with T2DM were included in the analysis (Fig. 1). Diabetes was defined as fasting plasma glucose ≥ 126 mg/dl or 2-hour plasma glucose ≥ 200 mg/dl after taking a 75-g oral glucose tolerance test using American Diabetes Association criteria^12^, or current treatment with oral antidiabetes drugs or insulin.

Our main outcome of interest was all-cause mortality. Death event was recorded annually. The baseline variables were obtained at the time of entry into the registry. Body mass index (BMI) was calculated as weight in kg divided by squared height in meters. Height and weight of patients were measured wearing light clothes and no footwear in the standing position while the shoulders were in the normal state using Seca stadiometer. The weight values were rounded to the nearest 0.1 kg. The baseline weight and height of patients were used to calculate their BMI. Resting systolic (SBP) and diastolic blood pressure (DBP)were measured after the patient seated for 5 minutes by using a calibrated mercury sphygmomanometer (Rester, Germany) with standard method recommended by the American Heart Association^13^. Blood pressure was measured twice on the same arm and the same patient position if blood pressure was high, and the mean value was recorded. Fasting plasma glucose was measured using the glucose oxidase method. Total cholesterol and HDL-cholesterol were measured by CHOD-PAP and triglyceride was determined by GPO-PAP methods. LDL-cholesterol was calculated using friedewald formula when total triglyceride was less than 400 mg/dL and non-HDL-cholesterol by subtracting HDL-cholesterol from the total cholesterol value.

Duration of diabetes was calculated by adding the duration of follow up for each case to the duration of diabetes at the baseline. Duration of diabetes at the baseline was estimated either from the date of the first abnormal laboratory report for diabetes or the date of the first diabetes-related treatment, whichever was earlier. Follow up duration was calculated from the date of examination at the baseline to the time of death, otherwise up to 2010.Life table is a key tool and a convenient method to compare the mortality and analyse death rates at various ages. Under the null hypothesis that patients with diabetes had a higher mortality risk than the general population, we used the national life tables for Iran in 2004^14^ to compare mortality rates of people with type 2 diabetes with the general population according to sex and age.

Laboratory analyses were carried out at the Isfahan Endocrine and Metabolism Research Center. The study was approved by the Medical Ethics Committee of the Isfahan University of Medical Sciences.

### Statistical analysis

The statistical analysis of the data was conducted with the STATA software version 11.0. Comparison of the baseline characteristics by participation status were performed by Student’s t-test. We estimated total, sex- and age-specific all-cause mortality rates (per 1000 person-years). In addition, for both males and females, we estimated mortality rates for five different diabetes duration (0–4, 5–9, 10–14, 15–19, and > = 20 years) for patients of all age groups and separately for patients aged < = 60 years and those aged >60 years. These rates were calculated as the number of subjects who died in each group, divided by the person-years of follow up. We calculated un-adjusted age-sex specific relative risk by dividing aforementioned rates per correspondent mortality rates in the general population. Life-years lost were estimated for each deceased person using age-group life expectancies reported for Iranian general population. We subtracted the age of death for each deceased person from his/her expected years of life. The national life tables for Iran in 2004 were used to estimate the relative risk and life-years lost^14^.

### Ethical approval

All procedures performed in studies involving human participants were in accordance with the ethical standards of the institutional and/or national research committee and with the 1964 Helsinki declaration and its later amendments or comparable ethical standards. The study was approved by the ethics Committee of the Isfahan University of Medical Sciences.

### Informed consent

Informed consent was obtained from all patients for participation and registration.

## Results

In total, 2451 patients with T2DM (993 (40.51%) men and 1458 (59.49%) women) were studied. Mean (SD) of diabetes duration was 7.8 (3.9) and median (Q1, Q3) of follow-up was 8 (5, 10) years. 732 (29.87%) of patients (406 (40.89%) men and 326 (22.36%) women (p < 0.001)) died during the follow-up.

Baseline characteristics by survival status are presented in Table 1. The values of some variables, including diabetes duration, SBP, DBP, FPG, LDL-cholesterol, HbA1c and smoking status, were higher in dead than live people. The proportion of patients treated with insulin, oral agents, diet and combination of insulin and oral agents were 10.6%, 64.5%, 22.4% and 2.4%, respectively. Dead people were more likely to be treated with insulin (20.3% vs. 6.9%), and less likely to receive oral agents (57.7% vs. 67.2%), dietary treatment (20.0% vs. 23.4%) and to be treated with combination of insulin and oral agents (2.1% vs. 2.6%) than live people.

**Table 1:** Baseline characteristics of patients with T2DM by survival status.

Variables		Total (n = 2451)	Alive (n = 1719)	Dead (n = 732)
		Mean or Number (SD or %)	Mean or Number (SD or %)	Mean or Number (SD or %)
Age (year) (n = 2451)		53.3 (10.1)	50.1 (8.8)	60.8 (8.9)
Age group(year)	≤ 60	1208 (49.3)	1047 (86.7)	161 (13.3)
	> 60	1243 (50.7)	672 (54.1)	571 (45.9)
Sex	Male	933 (40.5)	587 (34.2)	406 (55.5)
	Female	1458 (59.5)	1132 (65.9)	326 (44.5)
Diabetes duration (years) (n = 2451)		15.5 (8.0)	12.8 (6.5)	21.7 (7.8)
BMI (kg/m^2^) (n = 2369)		27.7 (4.5)	28.3 (4.4)	26.2 (4.4)
SBP (mmHg) (n = 2373)		127.1 (20.2)	123.8 (17.6)	135.6 (23.7)
DBP (mmHg) (n = 2372)		78.4 (12.3)	77.1 (11.9)	81.7 (12.6)
FPG (mg/dl) (n = 2408)		186.9 (71.6)	178.3 (66.9)	208.2 (78.2)
HDL-C (mg/dl) (n = 1964)		44.5 (11.4)	44.5 (11.3)	44.0 (12.5)
LDL-C (mg/dl) (n = 1802)		130.3 (42.4)	128.2 (40.7)	146.7 (50.8)
Non-HDL-C (mg/dl) (n = 1948)		174.6 (49.6)	172.5 (48.0)	190.0 (58.2)
HbA1c (%) (n = 1779)		8.4 (2.1)	7.5 (1.7)	10.1 (2.3)
Smoking (Yes) (n = 1934)		235 (12.2)	107 (8.5)	128 (19.1)
Treatment status	Insulin	255 (10.6)	118 (6.9)	137 (20.3)
	Oral agent	1545 (64.5)	1155 (67.2)	390 (57.7)
	Diet	537 (22.4)	402 (23.4)	135 (20.0)
	Insulin&oral agents	58 (2.4)	44 (2.6)	14 (2.1)

^a^P value based on Student’s T-test between total included participants and excluded participants, BMI: body mass index; SBP: systolic blood pressure; DBP: diastolic blood pressure; FPG: fasting plasma glucose, C: Cholesterol, TG: triglyceride, HDL: high density lipoprotein, LDL: low density lipoprotein.

The baseline characteristics by participation status can be found as Supplementary Table S1. Some variables (SBP, DBP, FPG, triglyceride and Hb_1_Ac) showed statistically significant differences between included and excluded patients, however, these differences were trivial and clinically negligible (Table S1).

The rate of mortality was increased with diabetes duration based on sexes and in 5-year intervals (Table 2). The mortality rates (95%CI) in all participants were 0, 6.6(4.3–10.3), 18.3(15.1–22.2), 43.8(38.3–50.2) and 74.3(67.3–82.0) per 1000 person-years for diabetes duration <5, 5–9, 10–14, 15–19 and ≥20 years, respectively. The mortality rates in males were higher than female patients in all categories. Overall mortality rates (95%CI) per 1000 person-years in men and women were 56.3(52.0–62.1) and 27.3(24.5–30.4), respectively. The patients were classified into two categories (age 60 years or less and more than 60 years) in order to control the age effect in assessing association between all-cause mortality rate and diabetes duration. The same results were obtained for both age categories (Table 2). Overall mortality rates in patients aged 60 years or less was.

**Table 2:** Sex- and age-specific all-cause mortality rates by diabetes duration in patients with T2DM.

Variable		Both sexes			Males			Females		
		Number	Deaths	MR (95% CI)	Number	Deaths	MR (95% CI)	Number	Deaths	MR (95% CI)
**All age groups**										
Diabetes duration	0–4 y	118	0	0.0	46	0	0.0	72	0	0.0
	5–9 y	493	20	6.6 (4.3–10.3)	180	9	8.0 (4.2–15.4)	313	11	5.8 (3.2–10.4)
	10–14 y	679	105	18.3 (15.1–22.2)	260	63	30.7 (24.0–39.3)	419	42	11.4 (8.4–15.5)
	15–19 y	525	211	43.8 (38.3–50.2)	203	111	65.8 (54.6–79.2)	322	100	32.0 (26.3–38.9)
	≥20 y	636	396	74.3 (67.3–82.0)	304	223	99.0 (86.8–112.9)	332	173	56.2 (48.4–65.2)
Total		2451	732	38.2 (35.5–41.1)	993	406	56.3 (52.0–62.1)	1458	326	27.3 (24.5–30.4)
**Age group ≤60 years**										
Diabetes duration	0–4 y	112	0	0.0	44	0	0.0	68	0	0.0
	5–9 y	366	4	1.9 (0.7–5.0)	130	2	2.6 (0.7–10.4)	236	2	1.5 (0.4–5.8)
	10–14 y	373	24	7.7 (5.2–11.5)	120	16	17.7 (10.8–28.9)	253	8	3.6 (1.8–7.3)
	15–19 y	215	51	25.2 (19.2–33.2)	69	28	47.1 (32.5–68.2)	146	23	16.1 (10.7–24.2)
	≥20 y	142	82	76.0 (61.2–94.4)	61	44	113.1 (84.2–142.0)	81	38	55.1 (40.1–75.7)
Total		1208	161	18.8 (16.1–21.9)	424	90	32.8 (26.6–40.3)	784	71	12.2 (9.6–15.4)
**Age group >60 years**										
Diabetes duration	0–4 y	6	0	0.0	2	0	0.0	4	0	0.0
	5–9 y	127	16	18.0 (11.0–29.3)	50	7	19.7 (9.4–41.4)	77	9	16.8 (8.7–32.3)
	10–14 y	306	81	30.9 (24.8–38.4)	140	47	40.9 (30.7–54.4)	166	34	23.1 (16.5–32.3)
	15–19 y	310	160	57.3 (49.1–66.9)	134	83	75.9 (61.2–94.2)	176	77	45.3 (36.2–56.7)
	≥20 y	394	314	73.8 (66.1–82.5)	243	179	96.1 (83.0–111.2)	251	135	56.5 (47.7–66.9)
Total		1243	571	54.0 (49.8–58.6)	569	316	70.8 (63.4–79.0)	674	255	41.7 (36.9–47.2)

MR: mortality rate; ^a^Rates were calculated per 1000 PY (person-years).

All-cause mortality rates by age at the end of study in women and men are presented in Table 3. The results showed that the mortality rate among patients with diabetes was higher than the general population in all age groups except for the age group of ≥75 years old. The relative risks (95%CI) of all-cause mortality in males vs. females with T2DM aged 45–49, 50–54, 55–59, 60–64, 65–69, 70–74 were [3.02(1.49–6.11) vs. 2.09(0.96–4.57)], [4.05(2.73–6.01) vs. 2.29(1.52–3.45)], [4.13(3.26–5.24) vs. 1.70(1.23–2.35)], [2.42(1.90–3.07) vs. 1.82(1.46–2.27)], [2.36(2.02–2.76) vs. 1.49(1.25–1.78)] and [1.71(1.50–1.95) vs. 1.04(0.88–1.23)] times more than the general population, respectively.

**Table 3:** All-cause mortality rates in Iranian patients with T2DM, compared to 2004 national life table.

Age at the end of the study	Number	Deaths	MR^a^	(95% Confidence Interval)	PMR^b^	Relative Risk (95% Confidence Interval)^c^
**Women**						
30–34	1	0	0.0	—	1.3	—
35–39	16	0	0.0	—	1.5	—
40–44	58	0	0.0	—	2.1	—
45–49	128	6	6.7	(3.0–14.9)	3.2	2.09 (0.96–4.57)
50–54	251	21	11.7	(7.6–18.0)	5.1	2.29 (1.52–3.45)
55–59	287	33	13.6	(9.7–19.2)	8.0	1.70 (1.23–2.35)
60–64	243	60	27.5	(21.3–35.4)	15.1	1.82 (1.46–2.27)
65–69	213	77	40.6	(32.4–50.7)	27.2	1.49 (1.25–1.78)
70–74	146	72	51.9	(41.2–65.4)	49.7	1.04 (0.88–1.23)
75–79	77	39	58.6	(42.8–80.3)	88.5	0.66 (0.53–0.82)
80–84	30	14	52.6	(31.2–88.9)	174.7	0.30 (0.20–0.44)
≥ 85	7	4	59.7	(22.4–159.1)	376.9	0.16 (0.08–0.30)
**Men**						
30–34	0	0	0.0	—	2.7	—
35–39	9	1	25.6	(3.6–182.0)	2.9	8.83 (1.39–56.05)
40–44	28	1	6.4	(0.9–46.1)	3.7	1.73 (0.25–11.86)
45–49	73	7	15.1	(7.2–31.7)	5.0	3.02 (1.49–6.11)
50–54	105	20	30.4	(19.6–47.2)	7.5	4.05 (2.73–6.01)
55–59	169	49	43.4	(32.8–57.4)	10.5	4.13 (3.26–5.24)
60–64	135	45	42.9	(32.0–57.5)	17.7	2.42 (1.90–3.07)
65–69	143	75	68.7	(54.8–86.1)	29.1	2.36 (2.02–2.76)
70–74	149	89	80.7	(65.6–99.3)	47.2	1.71 (1.50–1.95)
75–79	122	79	80.3	(64.4–100.1)	79.1	1.02 (0.89–1.16)
80–84	46	31	72.4	(50.9–103.0)	151.1	0.48 (0.39–0.59)
≥ 85	14	9	81.1	(42.2–155.8)	318.1	0.25 (0.17–0.37)

^a^Mortality rate calculated as the number of subjects who died at each group, divided by the person-years (PY) of follow up. Rates were calculated per 1000 PY.

^b^Population Mortality Rates, adopted from 2004 national life table for Iran, reported by Khosravi *et al*.^14^.

^c^It was calculated by this formula: $\mathrm{Ln}(\widehat{{\boldsymbol{RR}}})\pm {\boldsymbol{z}}\sqrt{\frac{({{\boldsymbol{n}}}_{1}-{{\boldsymbol{x}}}_{1})/{{\boldsymbol{x}}}_{1}}{{{\boldsymbol{n}}}_{1}}+\frac{({{\boldsymbol{n}}}_{2}-{{\boldsymbol{x}}}_{2})/{{\boldsymbol{x}}}_{2}}{{{\boldsymbol{n}}}_{2}}}$. Then we took the antilog (exp) of the lower and upper limits.

**Figure 2:**
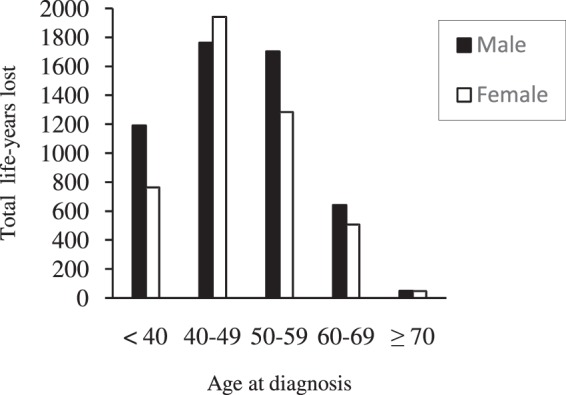
Total life-years lost for people with T2DM by age at diagnosis.

Mean (SD) years of life lost by age at diagnosis and sex are shown in Table 4. The youngest age group (<40 years old) had the highest years of life lost (20.2(7.1) for men and 21.8(5.4) for women), however with increasing age, it gradually declined (5.4(1.5) for men and 5.1(1.5) for women, aged 70–75 years old). We found that diabetes cuts life expectancy by 3.1(0.0) to 21.8(5.4) years according to age and sex. Men and women with diabetes lost an average of 13.2(6.3) and 13.9(6.0) life-years from the year of diagnosis, respectively (p = 0.101) (Table 4). The total years lost by age at diagnosis and sex are shown in Fig. 2. Considering we summed number of years lost for each age- and sex- specific group, values of y-axis have been increased to 2000 to cover all age- and sex- specific years lost.

**Table 4:** Mean life-years lost for Iranian patients with T2DM, compared to the general population using 2004 national life table.

Age at diagnosis	Male	Life-years lost^a^	Female	Life-years lost^a^	Total	Life-years lost^a^
	Number	Mean (SD)	Number	Mean (SD)	Number	Mean (SD)
<40	59	20.2 (7.1)	35	21.8 (5.4)	94	20.8 (6.5)
40–44	45	16.5 (6.6)	59	17.8 (5.2)	104	17.2 (5.8)
45–49	66	15.5 (5.2)	58	15.3 (5.1)	124	15.4 (5.1)
50–54	70	12.2(4.5)	56	12.9 (4.1)	126	12.5 (4.3)
55–59	76	11.1 (3.2)	50	11.3 (3.0)	126	11.2 (3.1)
60–64	44	8.7 (2.6)	37	9.4 (2.8)	81	9.0 (2.7)
65–69	36	7.2 (2.1)	22	7.2 (2.0)	58	7.2 (2.1)
70–74	8	5.4 (1.5)	9	5.1 (1.5)	17	5.3 (1.4)
≥ 75	2	3.1 (0.0)	0	—	2	3.1 (0.0)
Total	406	13.2 (6.3)	326	13.9 (6.0)	732	13.5 (6.2)

^a^Life-years lost were estimated for each deceased person by subtracting the age of death for each deceased person from his/her expected years of life.

## Discussion

In this observational study, 2451 patients with T2DM were followed for 18 years. We found, first, the patients with diabetes, aged 40–75 years old, according to sex and age, were at 1.04–4.13 times higher risk of mortality compared to the general population. Second, the excess mortality decreased with increasing age in both sexes compared to the general population. Third, the rate of mortality increased with diabetes duration in both sexes. Finally, men and women lost an average of 13.2(6.3) and 13.9(6.0) years from the age of diagnosis with diabetes, respectively. There is a gradual decline in years of life lost with increasing age of diabetes diagnosis.

The excess risk of mortality among people with diabetes has shown in previous studies^2,6,15^. These studies have also reported that relative risk of mortality tends to decline with increasing age. A Swedish national study showed that mortality rate increased between 100% to 200% among patients with T2DM, younger than 55 years old, compared to the general population; whereas the excess risk of death ranged from 30% to 40% among patients aged 65 to 74 years old^2^. A cohort study in Mexican adults reported that rate ratios for all-cause mortality in people with diabetes was 5.4 for 35–59 years old and 3.1 for60–74 years old patients^15^. Danish population with diabetes was at 1.5–2.5 times higher risk of mortality compared to the general population^6^. This risk decreased with age and at ages of more than 80 years, the mortality rate difference between the general population and patients with diabetes was small^6^. This decline of mortality rate with age might be caused by more sever effects of diabetes in younger people^16,17^. However, ageing also increases the risk of other life-threatening diseases in the general population^18^ whichmight increase the mortality rate in the older general population.

In the current study, the rate of mortality increased with diabetes duration. Our finding is consistent with previous studies^19–21^; In the Verona Diabetes Study (VDS), death rates rose in both sexes with increasing duration of diabetes^19^. In a cohort study, a10-yearlonger duration of diabetes was correlated with a 1.2–1.3 times increased risk of all-cause mortality in Australian patients with diabetes^20^. Another study in Mexico reported significantly increased rates of death from diabetes with duration of diabetes^21^. Longer duration of diabetes (equivalent to diagnosis of diabetes at younger age) increases the exposure to hyperglycaemia, which is correlated to an increased risk of complications^16,17,22^. Moreover, previousstudies^17,23^ have shown that early onset of T2DM is associated with more adverse cardiovascular risk profile. Prolong exposure to hyperglycaemia combined with the presence of multiple cardiovascular risk factors increases morbidity and mortality in patients with long duration of diabetes^22^.

Our study showed that the youngest age group (<40 years old) of patients with diabetes had the largest years of life lost (20.2 for men and 21.8 for women) and years of life lost was gradually declined with age (5.4 for men and 5.1 for women, aged 70–75 years old). We found that diabetes cut the life expectancy by 3.1 to 21.8 years according to age and sex with an average of 13.2 and 13.9 years in men and women, respectively. Different numbers of life-years lost have been reported for patients with diabetes by studies at various geographical area, racial background, sex and healthcare systems^7–9^. 6.10 years of life years lost is reported for Korean patients with diabetes in a cohort study, this number was declined with age^9^. Another study used NHIS data of the USA and found diabetes cut life expectancy by 3.30 to 18.74 years depending on age, sex, race, and BMI. Life years lost was also reported to decline with age^7^. A recent cohort study in England showed at age 40, white men and women with diabetes lost 5 and 6 years of life respectively, compared to those without diabetes^8^. The years of life lost in our study, were higher than previous reports. The early deaths might be the combination of several factors: the rapid changes in the lifestyle that led to reduced levels of physical activities; overconsumption of energy-dense food that results in obesity and young-onset diabetes^24^; late diagnoses of diabetes and the health systems that are not ready to provide optimal management to the increasing numbers of people with diabetes.

We have found a significantly higher mortality rate in men compared to women with diabetes. This is also occurred in the Iranian general population aged less than 70 years old^14^. This study reported all-cause mortality, not diabetes related mortality, and therefore, the higher rates of mortality in men, similar to the general population, might be due to other causes such as car or workplace accidents.

The present study has several strengths and limitations. This is the first retrospective database study, focusing on the mortality rate and the effect of diabetes on the expected life years among Iranian people with long-term follow-up (18 years). The limitations of the current study include: (i) referral bias in the clinic-based studies is likely to produce higher mortality rates than in the community-based studies; (ii) we have analysed the data for patients with T2DM with follow-up visits and clear outcome in 2010. Those patients with irregular visits and unclear outcome were excluded which may cause a selection bias. However, we compared the baseline characteristics between the included and excluded groups, which indicated clinically trivial differences and therefore, we expect this bias to be negligible; (iii) use of local population without diabetes as the comparison group can minimize the bias in the estimation of mortality ratios. However, the data for mortality rates in the local population without diabetes was unavailable; (iv) the data for the mortality caused by cardiovascular disease or cancer and information about some medications such as anti-hypertensive and lipid-lowering drugs at the baseline were unavailable.

In conclusion, our study showed people with diabetes were at higher risk of death and lower life expectancy compared to the general population. The young people with diabetes lost more years of life compared to people who diagnosed with diabetes at the older ages. Therefore, more healthcare attention should be paid to these patients to improve their life expectancy.

## Supplementary information

Baseline characteristics of patients with T2DM by participation status.

## Data Availability

The datasets generated during and/or analysed during the current study are available from the corresponding author on reasonable request.
