# Supplementary material for: Diabetes and all-cause mortality, a 18-year follow-up study
Source: Sci Rep. 2020 Feb 21;10:3183. doi: 10.1038/s41598-020-60142-y (PMC7035261; doi:10.1038/s41598-020-60142-y)
Supplement: Supplementary file 1 — Baseline characteristics of patients with T2DM by participation status. [file 41598_2020_60142_MOESM1_ESM.pdf]

Diabetes and all-cause mortality, a 18-year follow-up study

Rezvan Salehidoost[1], Asieh Mansouri[2], Massoud Amini[3]*, SimaAminorroaya Yamini[4], Ashraf Aminorroaya[3]*

Isfahan Endocrine and Metabolism Research Center, Isfahan University of Medical Sciences, Isfahan, Iran

[1]Assistant professor, Isfahan Endocrine and Metabolism Research Center, Isfahan University of Medical sciences, Isfahan, Iran

[2]PhD of Epidemiology, Hypertension Research Center, Cardiovascular Research Institute, Isfahan University of Medical Sciences, Isfahan, Iran

[3]Professors of Endocrinology, Isfahan Endocrine and Metabolism Research Center, Isfahan University of Medical Sciences, Isfahan, Iran

[4]Reader, Department of Engineering and Mathematics, Sheffield Hallam University, Sheffield, S1 1WB, UK.

[*]Corresponding author: Prof.Ashraf Aminorroaya and Prof.MassoudAmini

Table S1. Baseline characteristics of patients with T2DM by participation status

Variables	Included participants (n=2451)	Excluded participants (n=9246)	P-value[a]
	Mean (SD)	Mean (SD)	
BMI(kg/m^2)	27.7 (4.5)	27.7 (4.6)	0.492
SBP (mmHg)	127.1 (20.2)	125.5 (20.0)	< 0.001
DBP (mmHg)	78.4 (12.3)	77.0 (12.4)	< 0.001
FPG (mg/dl)	186.9 (71.6)	199.6 (73.5)	< 0.001
Total-C (mg/dl)	219.9 (50.0)	221.0 (51.0)	0.251
TG (mg/dl)	219.3 (146.1)	229.7 (155.7)	0.006
HDL-C (mg/dl)	44.5 (11.4)	45.5 (11.9)	0.267
LDL-C (mg/dl)	130.3 (42.4)	127.3 (42.2)	0.261
HbA1c (%)	8.4 (2.1)	8.6 (2.4)	< 0.001

[a]P value based on Student's T-test between total included participants and excluded participants, T2DM: type 2 diabetes mellitus, BMI: body mass index; SBP: systolic blood pressure; DBP: diastolic blood pressure; FPG: fasting plasma glucose, C: Cholesterol, TG: triglyceride, HDL: high density lipoprotein, LDL: low density lipoprotein
